# Impact of physician–patient relationship training on medical students’ interpersonal skills during simulated medical consultations: a cross-sectional study

**DOI:** 10.1186/s12909-022-03171-7

**Published:** 2022-02-22

**Authors:** Lucie Bosméan, Philippe Chaffanjon, Alexandre Bellier

**Affiliations:** 1 Department of Family Medicine, University of Grenoble Alpes, Grenoble, France; 2 School of Medicine, University of Grenoble Alpes, Grenoble, France; 3 Clinical Epidemiology Unit, Grenoble Alpes University Hospital, CS10217, Cedex 09, 38043 Grenoble, France; 4 Computational and Mathematical Biology Team, TIMC UMR 5525, CNRS, University of Grenoble Alpes, Grenoble, France

**Keywords:** Clinical competence, Health education, Interpersonal skills, Medical students, Simulation

## Abstract

**Background:**

In medicine, the patient-centered approach is based on interpersonal skills, including communication, structuring the medical interview, and empathy, which have an impact on health professionals’ interpersonal relationships and the quality of care. Training courses on this issue are therefore being developed in universities. We hypothesized that specific training courses in the physician–patient relationship could improve interpersonal skills among medical students during simulated consultations and the immediate satisfaction of standardized patients.

**Methods:**

This cross-sectional study enrolled fourth-year medical students who participated in a simulated medical consultation session with standardized patients. The evaluation of interpersonal skills was carried out using the Four Habits Coding Scheme, producing a synthetic score out of 115 points used as the primary endpoint. Some students benefited from the training courses offered by the university or by other organizations, mainly based on communication, active listening, or patient-centered approach. A comparison was made with students from the same graduating class who had not received any training.

**Results:**

The analysis of the primary endpoint showed a difference of 5 points between the group of students who had attended at least one training course and those who did not (*p* = 0.001). This difference was even more marked when the students had completed several training courses, up to 14 points higher with three training courses (*p* = 0.001), each with positive results in different areas of the care relationship.

**Conclusions:**

Physician–patient relationship training currently provided in initial education appears to be effective in improving interpersonal skills. A repetition of this training is necessary to increase its impact.

## Background

In medicine, the patient-centered approach is based on interpersonal skills, including communication, structuring the medical interview, and empathy, which have an impact on health professionals’ interpersonal relationships and the quality of care [1, 2]. Interpersonal skills are defined as the presence of verbal and nonverbal behaviors in the context of personal interactions with the patient or the patient’s family [3]. French universities are starting to offer training courses on this specific issue. Indeed, interpersonal skills are one of the essential skills to be taught in medical curricula and are among the most appreciated by patients [4–6]. They increase the quality of care and help decrease human or economic costs due to adverse events [7]. Finally, it has been shown that communication skills tend to decline over time unless they are regularly recalled and practiced [8]; the same applies to empathy, which also decreases over time – a finding that has been validated since 2010 [4].

In the interest of the patient and the quality of care, it is therefore essential to consider whether training can help halt this decline, and to reflect on the personal benefit to the practitioner. Several studies have indicated that communication skills can be taught and learned in both simulated and real clinical settings [9–11]. It was shown that short-term training focusing on interpersonal skills can lead to a significant change in behavior (*p* = 0.010) and long-term self-efficacy (*p* = 0.042) for senior physicians [12]. In a review of the literature, it was reported that educational interventions can also be effective in maintaining and reinforcing empathy among medical students from the beginning of training, with a mean effect of 0.23 on the Medical Education Research Study Quality Instrument score [13].

It is sometimes difficult to demonstrate the effectiveness of these programs on physician competencies, and generally only one component of the physician–patient relationship is assessed, such as empathy [14] or communication [9]. Another gap highlighted by a recent study is the difficulty in demonstrating a transfer of skills from physician to patient [15]. Few studies have been able to show an impact on the patient [16, 17], and only one randomized trial [17] focused both on the impact of training on interpersonal skills for the hospital as a whole and on the impact for the patient in terms of immediate satisfaction. Furthermore, the population studied was largely physicians, more often at the hospital, and less often medical students. Finally, the retention over time of the skills acquired is a crucial point. It seems that the effect of training, in this case a multifaceted program inspired by the Kaiser Permanente [12], on interpersonal skills may persist in the long term. However, few studies have measured the medium- or long-term retention of skills acquired through training, and to our knowledge, no studies have examined the long-term retention of interpersonal skills of medical students in training targeted at interpersonal skills.

In order to carry out such evaluations in medical consultation, it is necessary to have standardized, valid, and reliable instruments [5]. On the basis of its satisfactory psychometric properties, several entities in numerous countries use the Four Habits Coding Scheme (4-HCS) [1] to assess interpersonal skills (Empathy, Interview Structuring, and Communication) [16, 17]. This scale has four sub-sections: Involvement from the beginning (Habit 1), Getting the patient’s point of view (Habit 2), Demonstrating empathy (Habit 3), and Involvement until the end (Habit 4). For each of the 23 items, the rating uses a 5-point Likert scale. By adding up the points, the scale produces a composite score from 23 to 115 points, as well as specific scores for each dimension. The 4-HCS has been used with thousands of physicians worldwide [12, 17–20] and a French translation has been validated [5].

The main objective of our study was therefore to measure the impact that training in physician–patient relationships can have on the interpersonal skills of medical students during simulated consultations. The secondary objectives were to compare the effectiveness of the different training courses on improving the students’ interpersonal skills and to evaluate the immediate satisfaction of standardized patients at the end of the consultations.

## Methods

### Study design

We conducted a cross-sectional study in the field of medical education, with a prospective recruitment design. The study took place from October 2019 to January 2020 at Grenoble Alpes University Hospital. We followed the international STROBE guidelines for this study [21].

### Participants

Fourth-year medical students enrolled in the Faculty of Medicine of the University of Grenoble Alpes during the 2019–2020 academic year were eligible to participate in the study. Students were required to participate in the practice as part of their curriculum. Each medical student participated in a single simulated consultation. At the time of the evaluation, each student had the same clinical background. We excluded students who were not available at the time of their convocation.

### Data collection

The requested exercise consisted in carrying out a consultation limited to questioning with the participation of actors from the university’s Department of Performing Arts, as standardized patients [2]. The objective was to place the medical student in a realistic clinical situation (consultation for common pathologies, without emergency criteria and accessible to outpatients, as in a general medicine consultation) with typical stereotyped patients. This exercise was the first simulated consultation for these students. All simulated consultations were video-recorded, and the videos were stored in a database on a secure cloud for later remote access [2]. The video made it possible to perform one or more evaluations after the consultation, without the need for the physical presence of an evaluator during the consultation, which could affect the medical student’s behavior [22]. Each consultation was evaluated by one of three physicians with experience in interpersonal skills assessment. These physicians evaluated the videos independently of each other during the same period. Evaluators completed an online form in which all items on the scale were mandatory, thus there could be no missing data.

The evaluation of interpersonal skills was made using the 4-HCS [2] scale, which resulted in a total score and four sub-scores corresponding to each dimension (Habit 1, Habit 2, Habit 3, and Habit 4). The 4-HCS score was the primary endpoint of the study. The evaluation of the videos and the calculation of the 4-HCS score were performed blind to the type of training undertaken by the student. Performing Arts students were asked to complete an evaluation of service consumers and health systems using a visual analog scale (VAS) rated from 0 to 10: “Using this visual analog scale, where this end represents the worst possible doctor and the other end represents the best possible doctor, please rate this doctor by placing a cross corresponding to how you feel...” This scale was used to measure overall patient satisfaction [12], which was used as a secondary endpoint for evaluating interpersonal skills.

### Training courses

Our students were able to follow various short-term training courses on a voluntary basis to improve their interpersonal skills. Some of the courses were academic, others not, but we chose to evaluate all of them. Each course could be followed only once by a student. The first course, an academic training course in communication, was given by the Faculty of Medicine, in small groups (15 students) in the form of a 3-h interactive course: exchanges, role plays, and viewing of consultation examples. It dealt with ethics, the legislative framework of medical practice, and medical communication according to the Four Habits Model developed by the Kaiser Permanente Institute [16]. The course was given on an experimental basis 1 year before the simulated consultation exercise to approximately one third of the students in the class. The second course, peer training, was given by peers trained in active listening and medical communication, and took place in small groups (15 students) in the form of a 1-day interactive course that involved role-playing. It was based on Rogers’s active listening, emotional intelligence, and different communication profiles/channels. It was given 1 year before the simulated consultation exercise. The third training course, given by an association, was carried out by a psychologist in small groups (15 students) in the form of a 4-day interactive course: exchanges, work on photography, and role-playing. It was based on active listening according to Rogers and was given 2 years before the simulated consultation exercise. Finally, the fourth training set included training not mentioned above and was carried out by the students in a framework other than that of the university, having a link with medical communication. This included training in transactional analysis, bereavement support, medical communication courses in midwifery school, non-compulsory university teaching on speaking and listening in care, training in difficult announcements, training in team management, and training in support for children with disabilities.

Before the simulated consultations, the students were asked about their participation in one of these training courses conducted on a voluntary basis. All the training sessions were held more than 1 year before the simulated consultations and the evaluation focused on the long-term retention of the skills acquired. After giving information about the study and obtaining consent, the student volunteers were recruited and divided into two groups: a training group and a control group. In the training group, the students had partaken in at least one of the aforementioned training courses, while the students in the control group had not received any specific training in caregiver relationship skills. Thanks to the participation of all students, no data were missing.

### Statistical methods

Calculating the number of participants required was based on the study by Gulbrandsen et al. [12], which described an improvement in the 4-HCS score from 58.8 to 62.9 points after 20 h of specific training (standard deviation = 10). With an alpha risk of 5% and a power of 80%, the minimum number of participants was 95. Inclusion characteristics were described for the entire sample and for each training received. Univariate analyses were performed to compare student characteristics with the 4-HCS score using Student’s *t* test or a Mann-Whitney test depending on the distribution of the quantitative variable, as well as an ANOVA or Kruskal-Wallis test and a Pearson or Spearman correlation test depending on the validity conditions. The primary endpoint was analyzed using a multivariate analysis with linear regression model. In the case of an association of less than 0.20 between a measured characteristic and the 4-HCS score, the analysis was performed by introducing this variable as a fixed-effect covariate into the model. This adjustment was carried out on the standardized patient criteria (*p* = 0.031), on the day of the simulated consultation (*p* = 0.185), and on the number of training sessions (*p* = 0.192). The secondary endpoint was analyzed using linear regression models, following a similar strategy. The statistical significance level was set at 0.05 in the two-tailed situation for all comparison tests. No *p*-value adjustments were expected. Statistical analyses were performed using the software RStudio (Version 1.0.143©).

### Ethics

Students were given information about the research in person and were included only after signing an individual consent form and image rights. Ethics committee approval was granted on 22th February 2021, by the regional ethics committee: *Comité d’Ethique du Centre d’Investigation Clinique de Clermont-Ferrand* (IRB 00005891).

## Results

**Fig. 1:**
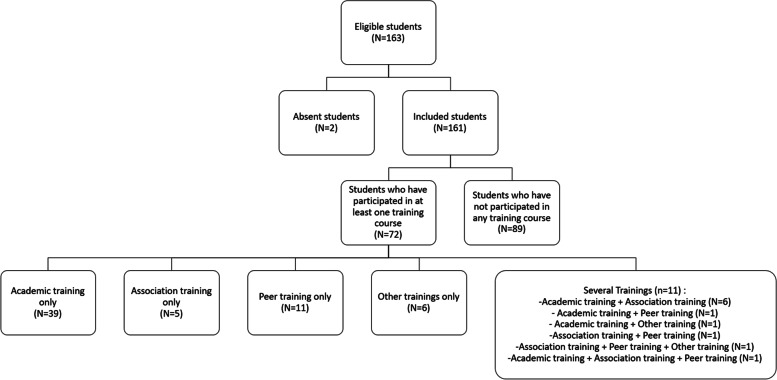
Flow chart of the study sample

Our sample consisted of 163 students who were invited to participate in the simulation of medical consultations. Two students were absent and no student refused to participate. Thus, we recorded and analyzed 161 videos of simulated consultations (Fig. 1). The average age of the students was 21 years and 69% were female (*N* = 112). A total of 72 students (44.7%) participated in interpersonal skills trainings: 48 students (29.8%) participated in the academic training, 14 (8.6%) in the training by an association, 15 (9.3%) in the peer training, and eight (4.9%) in other training as previously defined. Overall, 61 students (37.8%) participated in only one type of training.

Analysis of the main outcome (i.e., 4-HCS synthetic score) showed a difference of 6.7 points between the group of students with at least one training course and those without, with an average of 85 points (SD = 17.2) for all the students (Table 1). In multivariate analysis, the difference between the two groups of students was statistically significant (*p* = 0.001). It should be noted that this difference was even more marked when the students had completed several courses. Without training, students had a mean score of 82 points/115 (SD = 18.7), whereas after three different training courses the mean score was 109 points/115 (SD = 6.36). The difference in the 4-HCS score depending on the number of training courses taken was statistically significant (*p* < 0.001).

**Table 1:** Evaluation of the impact of the different training courses on the 4HCS (Four Habits Coding Scheme) and the AVS (analog visual scale)

Training	Outcomes	Intervention group Mean (SD)	Control group Mean (SD)	Unadjusted ***p***-value	Adjusted ***p***-value
All training combined (*N* = 72)	4HCS (/115)	88.7 (14.3)	82.0 (18.7)	0.060	**0.001**
	AVS (/10)	7.91 (1.54)	7.84 (1.33)	0.661	0.779
Academic Training (*N* = 48)	4HCS (/115)	88.6 (14.3)	83.5 (18.1)	0.080	**0.002**
	AVS (/10)	7.83 (1.53)	7.89 (1.39)	0.821	0.719
Association Training (*N* = 14)	4HCS (/115)	87.5 (17.7)	84.8 (17.2)	0.573	0.296
	AVS (/10)	8.04 (1.71)	7.86 (1.40)	0.656	0.574
Peer Training (*N* = 15)	4HCS (/115)	90.4 (15.3)	84.5 (17.3)	0.218	**0.031**
	AVS (/10)	8.07 (1.87)	7.85 (1.39)	0.587	0.495
Other training(*N* = 8)	4HCS (/115)	93.2 (13.0)	84.3 (17.4)	0.075	**0.007**
	AVS (/10)	8.04 (1.84)	7.86 (1.39)	0.664	**0.045**

When considering each training independently, there was a 5.1-point increase in the 4-HCS score for academic training (*p* = 0.002), a 5.9-point increase for peer training (*p* = 0.031), and an 8.9-point increase for other training (*p* = 0.007) (Table 1).

Analyzing the 4-HCS score according to the 4 Habits, several training courses were found to have an impact on Habit 1 with a 0.7-point increase in the score on this dimension (*p* = 0.047) for academic training and a 2.1-point increase (*p* = 0.025) out of 30 points for peer training. Academic training increased the score for Habit 2 by 1.1 points (*p* = 0.002) out of 15 points. For Habit 3, academic training increased by 1.2 points (*p =* 0.004) and other training by 2.3 points out of 20 points (*p* = 0.007). Finally, for Habit 4, the increase in score was 2.2 points (*p* = 0.005) for academic training and 2.7 points (*p* = 0.009) for other training (Table 2).

**Table 2:** Evaluation of the impact of the different training courses on the components of the 4HCS (Four Habits Coding Scheme): getting involved from the beginning (Habit 1), getting the patient’s point of view (Habit 2), showing empathy (Habit 3), and being involved until the end (Habit 4)

Training	Assessment	Training (Mean and standard deviations)	Training not taken (Mean and standard deviations)	Unadjusted ***p***-value	Adjusted ***p-***value
All training combined (*N* = 72)	Habit 1 (/30)	22. 0 (3.51)	21.0 (4.51)	0.171	**0.016**
	Habit 2 (/15)	12.3 (2.38)	11.2 (2.91)	**0.020**	**< 0.001**
	Habit 3 (/20)	15.0 (3.78)	14.0 (4.25)	0.146	**0.002**
	Habit 4 (/50)	39.0 (6.72)	36.7 (8.69)	0.078	**0.004**
Academic Training (*N* = 48)	Habit 1 (/30)	21.9 (3.40)	21.2 (4.47)	0.325	**0.047**
	Habit 2 (/15)	12.4 (2.29)	11.3 (2.89)	**0.021**	**0.002**
	Habit 3 (/20)	15.2 (3.75)	14.0 (4.20)	0.091	**0.004**
	Habit 4 (/50)	39.1 (6.94)	36.9 (8.44)	0.117	**0.005**
Association Training (*N* = 14)	Habit 1 (/30)	21.6 (4.34)	21.4 (4.18)	0.819	0.830
	Habit 2 (/15)	12.4 (2.47)	11.6 (2.79)	0 .302	0.151
	Habit 3 (/20)	15.2 (3.47)	14.3 (4.15)	0.433	0.252
	Habit 4 (/50)	38.3 (8.84)	37.5 (8.02)	0.739	0.303
Peer Training (*N* = 15)	Habit 1 (/30)	23.3 (3.75)	21.2 (4.19)	0.089	**0.025**
	Habit 2 (/15)	12.2 (2.67)	11.6 (2.77)	0.407	0.060
	Habit 3 (/20)	15.2 (4.10)	14.3 (4.10)	0.433	0.115
	Habit 4 (/50)	39.8 (6.28)	37.4 (8.20)	0.289	0.059
Other Training (*N* = 8)	Habit 1 (/30)	22.8 (3.70)	21.3 (4.21)	0.218	0.057
	Habit 2 (/15)	12.8 (1.57)	11.5 (2.82)	0.097	0.050
	Habit 3 (/20)	16.5 (3.43)	14.2 (4.10)	**0.048**	**0.007**
	Habit 4 (/50)	41.0 (7.20)	37.3 (8.09)	0.113	**0.009**

Concerning patient satisfaction at the end of the consultation, this trend was confirmed with an increase of 0.22 points out of 10 points for peer training, 0.18 for association training (*p* = 0.495 and *p* = 0.574, respectively), and 0.18 points for other training (*p* = 0.045) (Table 1). The mean score for all students was 7.87 (SD = 1.43).

## Discussion

The training in the physician–patient relationship improved the level of interpersonal skills as evaluated by the 4-HCS score: academic training, training given by peers, and training given in a private setting on the student’s own initiative. In addition, these short courses had a significant cumulative effect: the more courses taken by the students, the more their interpersonal skills improved.

Although the results were positive, we noted that the results differed according to the type of training taken. The interpersonal skills developed were not always the same and of the same intensity, due to the multidimensional aspect of these skills. Interpersonal skills encompass respectful attitude, attention paid to the patient, being personally present in the moment with the patient, interest in patient values and concerns, and real-time adjustment of the relationship [23]. It therefore seemed relevant to examine the mechanisms behind these differences in improvement, such as the content of the training courses and their duration. A preferred approach would be that they act not only on the communication factor in the physician–patient relationship, but also on the structuring of the interview and the empathy expressed by the health professionals. Depending on the aspect of interpersonal skills addressed, it would thus be possible to observe a difference in the increase in the specific scores of each Habit. This progression was observed more than 1 year after the training sessions, and depending on the training sessions carried out, the improvement could be felt by the patients during the simulation exercises. The evaluation of immediate satisfaction showed encouraging results, despite the fact that students have little clinical experience in the fourth year of medical studies. Students’ satisfaction with the exercise could explain their full participation in the questionnaires. This questionnaire was short and had to be filled out in the continuity of the simulation, which allowed us to have no missing data.

Firstly, we were pleased with the positive results on the interpersonal skills for all the training courses. Evidence is accumulating that interpersonal skills can be acquired and improved through teaching and practice-based training for medical students [24, 25], whereas several studies have failed to demonstrate the effectiveness of training courses on these skills [26–29]. It may be interesting to ask why and how training delivered at the beginning of the medical curriculum works so well, whereas the effect obtained in continuing education is generally only moderately satisfactory [30].

Indeed, the question of temporality is also important, since the impact of training tends to diminish over time [26, 31, 32]. However, and despite the very short training courses carried out at least 1 year before our study, only the training offered by an association did not show any improvement in the interpersonal skills. However, this training took place more than 2 years before the simulated consultations (i.e., during the pre-clinical phase of the medical curriculum), which may explain its more subtle effects. The effect tested was therefore a long-term improvement effect. Internal medicine clerkship performed by medical students — between the training and the simulated consultation — include other training components than clinical reasoning such as the opportunity to acquire experience in communication with real patients and to strengthen previously acquired interpersonal skills.

A limitation of this study was that we analyzed the practices of small groups of students in the subgroup analyses. Moreover the voluntary participation of the students in the trainings could be a bias, increasing the interest regarding the communication, the level of interpersonal skills expected is higher. The majority of students were volunteers to participate in the trainings. The lack of trainers did not allow to include all student volunteers in the different trainings.

The originality of this controlled study stems in particular from the heterogeneity of the training courses evaluated: the forms differed between courses provided by the medical faculty, courses offered by peers, or courses taken by professionals on their own initiative. The originality of this study is reinforced by the fact that the evaluation of the training impact on interpersonal skills was compared with pre-identified psychosocial risk factors. Finally, the evaluation of interpersonal skills was carried out during simulated consultations in order to be as close as possible to real-life practice conditions.

Concerning the impact of these training courses, we observed differences between the programs studied. Some of the training favored content focusing on ethics and the deontological framework and others focused on emotional experience, while some were integrated into the university curriculum and others were extra-curricular. However, the importance of the content of these programs and the environment seems to diminish with repetition. Thus, the best results were seen with the greatest number of training courses taken, even if the courses are heterogeneous. Thus, it seems that the number of training courses attended, and the regularity of these courses is more important than having sessions with standardized content. Whatever the combination of training, the effect on interpersonal skills was increased.

Research into the factors facilitating the integration of interpersonal skills in clinical practice is indispensable, as is the development of these training courses on interpersonal skills. In addition to reducing medical errors and enhancing patient safety, a beneficial effect of communication education is to increase students’ self-confidence over the long term, which contributes to maintaining good mental health, especially for individuals working in stressful environments, such as medical students [15, 33, 34]. A recent meta-analysis reported a low level of evidence for studies comparing a communication skills intervention to a control group for improving medical students’ interpersonal skills on only 15 studies [35]. The majority of the included studies had small group workshops (less than 70 participants) and had little to no effect on skills [35]. Effects on longer-term behaviour change are rarely studied and our study reports some evidence. Studies with a rigorous design seem to be needed to evaluate the long-term effects of different educational approaches in a standardized way.

## Conclusions

Using simulation exercises of medical consultations and a standardized evaluation of the interpersonal skills of medical students, we have shown the value of integrating training in the caregiver–patient relationship into the medical curriculum. Indeed, the impact of these training courses on students’ interpersonal skills is significant, even after more than 1 year, even if they are given early in the curriculum, and even if their content is heterogeneous. The training courses are all the more effective if they are repeated. This study has potential implications for optimizing interpersonal skills training and assessment as part of the medical curriculum.

## Data Availability

The datasets used and/or analysed during the current study are available from the corresponding author on reasonable request. The dataset used is not publicly available, as it contains links to video-recorded consultations that can identify the students involved in the study.

## Abbreviations

4-HCS: Four Habits Coding Scheme
SD: Standard Deviation
